# Complementary role of magnetic resonance imaging after ultrasound examination in assessing fetal renal agenesis: a case report

**DOI:** 10.1186/1752-1947-8-96

**Published:** 2014-03-12

**Authors:** Tomasz Gęca, Arkadiusz Krzyżanowski, Aleksandra Stupak, Anna Kwaśniewska, Tomasz Pikuła, Radosław Pietura

**Affiliations:** 1 Department of Obstetrics and Pathology of Pregnancy, Medical University of Lublin, 16 Staszica Street, 20-081 Lublin, Poland; 2 Department of Diagnostic Imaging, SPSK1 Lublin, 16 Staszica Street, 20-081 Lublin, Poland

**Keywords:** Magnetic resonance imaging, Fetus, Renal agenesis

## Abstract

**Introduction:**

Ultrasonography is used routinely during pregnancy to screen and detect fetal abnormalities. However, there are some conditions like anhydramnios (a prevalent state in renal agenesis) or maternal obesity that may limit the diagnostic accuracy of ultrasonography. Magnetic resonance imaging has proven to be useful when ultrasound alone is insufficient to make a correct diagnosis.

**Case presentation:**

We present the case of a 22-year-old Caucasian woman who was admitted to our unit at the 26th week of gestation for a detailed anatomy scan. Anhydramnios and failure to visualize the kidneys, bladder and renal vessels were confirmed with the use of sonography in our department. Since the lack of amniotic fluid limited the acoustic window for fetal ultrasonography, a magnetic resonance imaging scan was requested to confirm suspected renal agenesis. A fetal magnetic resonance imaging scan was performed and confirmed the suspected diagnosis. A baby boy was born by breech vaginal delivery after spontaneous onset of labor at the 34th week of gestation. The boy weighed 1690g, with Apgar scores of 6 and 4 at two and five minutes respectively, and died one hour after delivery. The diagnosis of bilateral renal agenesis was confirmed on autopsy.

**Conclusions:**

The aim of this study was to evaluate the potential contribution of magnetic resonance imaging in diagnostic procedure after inconclusive ultrasound examination during the assessment of fetal urinary tract abnormalities in the third trimester.

## Introduction

Undoubtedly, ultrasonography (USG) is an ideal imaging procedure during pregnancy. It is noninvasive, inexpensive, with no radiation risk and provides an opportunity to visualize the fetus. Sometimes ultrasound examination might be hampered by maternal obesity, oligo/anhydramnios, fetal position and reverberation caused by bones. When USG is unable to provide a definitive diagnosis, further investigation with more sophisticated methods is necessary. One of these methods is magnetic resonance imaging (MRI), which plays an increasingly important role in fetal visualization. MRI of a human fetus was first described in 1983 [1]. Initial attempts to use MRI in obstetrics were limited by fetal movement, despite pharmacological immobilization of the fetus [2,3]. Currently, the use of direct fetal paralysis is strongly discouraged. Some authors recommend pre-procedure maternal sedation in order to decrease fetal movements [4]. The development of ultrafast imaging techniques such as half-Fourier acquisition single-shot turbo spin-echo (HASTE), and echo planar imaging (EPI) decrease the duration of examination to 20 to 30 minutes. The most common indication for fetal MRI is not only the assessment of central nervous system abnormalities but also anomalies in fetal chest and abdomen [1,5]. According to the white paper on MRI safety, issued by the American College of Radiology, fetal MRI can be performed at any stage of pregnancy [6]. In the third trimester the lateral decubitus position is preferred to avoid inferior vena cava syndrome. It is recommended that informed consent be obtained from all pregnant women before an MRI examination [6]. In spite of the fact that MRI provides more anatomical details, it is more expensive than USG, not portable, and less available. In contrast to USG, it is an operator-independent technique.

The administration of contrast media during pregnancy is still controversial. Gadolinium, which is classified as a category C drug by the Federal Drug Administration (FDA), crosses the placenta, and is excreted by the fetal kidneys into the amniotic fluid. The recommendations of the American College of Radiology Guidance Document for safe MR practices state that intravenous gadolinium administration should be avoided during pregnancy [7].

Our aim was to assess the role of MRI as a complementary diagnostic tool in the absence of conclusive sonographic findings on the basis of a case study of anhydramnios in a 22-year-old pregnant woman.

## Case presentation

**Figure 1:**
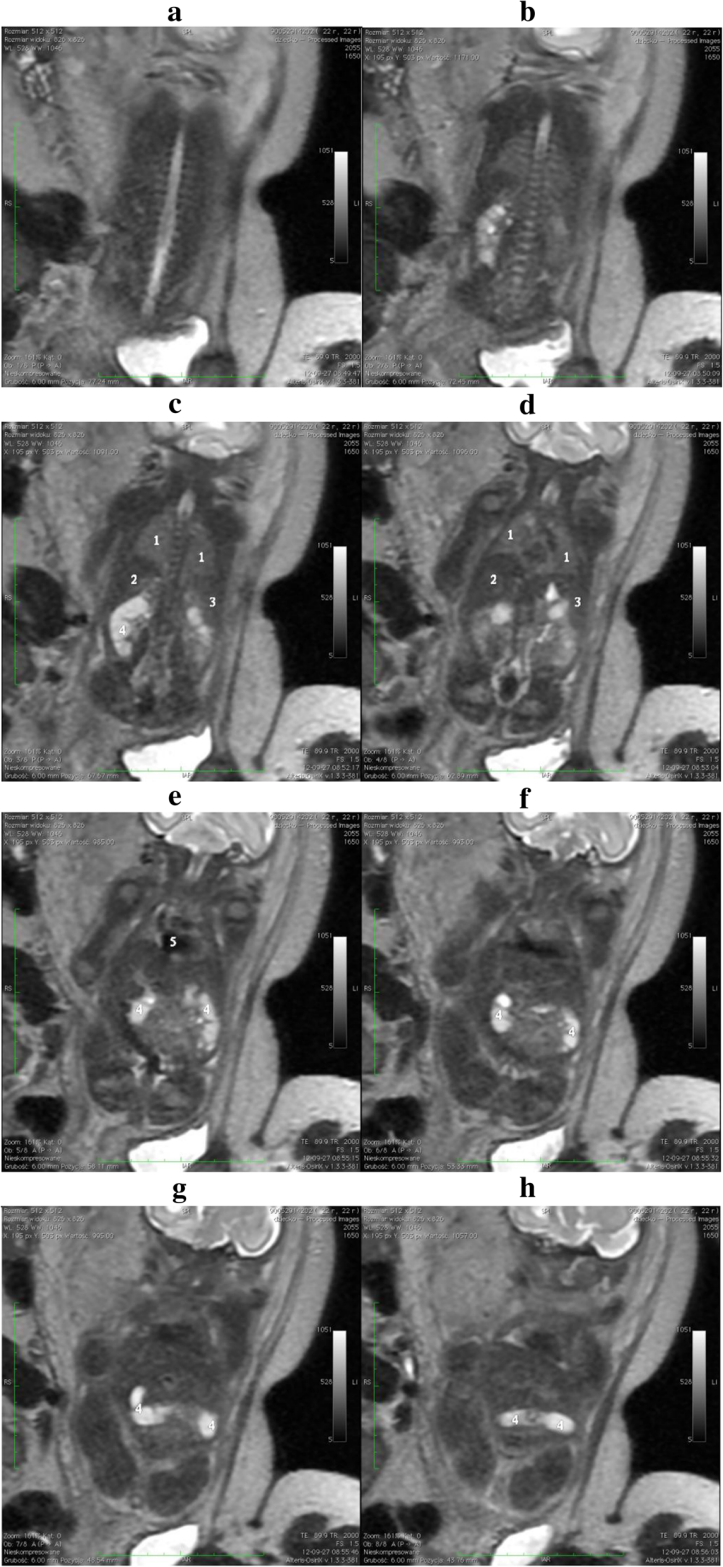
**Images of the coronal plane from the dorsal to ventral side of the fetus (a-h).** Single-shot fast spin-echo sequences, T2-weighted images. 1, lung; 2, liver; 3, spleen; 4, intestine; 5, heart.

A 22-year-old Caucasian woman, gravida 3 para 2, was admitted to our unit at the 26th week of gestation for a detailed anatomy scan. Her pregnancy had been uncomplicated and an ultrasound examination at the 12th week of gestation had not revealed any anomaly. Fetal growth and the amniotic fluid volume were normal. The transabdominal ultrasound examination at the 22nd week of gestation revealed anhydramnios, which lead to the patient being referred to our clinic. A detailed transabdominal ultrasound examination was performed using Voluson E8 equipment with a 5.0MHz convex probe (GE Healthcare, Little Chalfont, UK). Anhydramnios and failure to visualize the kidneys, bladder and renal vessels were confirmed with the use of sonography in our department. Since the lack of amniotic fluid limited the acoustic window for fetal USG, an MRI scan was requested to confirm suspected renal agenesis. A fetal MRI scan was performed two weeks later at the 28th week of gestation and confirmed the suspected diagnosis (Figure 1). No other anomalies were detected. MRI was performed using a General Electric Optima 360 1.5T scanner (GE Healthcare). Single-shot fast spin-echo sequences (SSFSE) were used, obtaining T2-weighted images in coronal, axial and sagittal planes. The MRI examination was well tolerated by our patient, and fetal movements did not alter the image quality, even though no maternal sedation was used.

A baby boy was born by breech vaginal delivery after spontaneous onset of labor at the 34th week of gestation. The boy weighed 1690g, with Apgar scores of 6 and 4, at two and five minutes, respectively. There was a respiratory effort observed and his heart rate was 50 to 60bpm. The parents requested no resuscitation, and the newborn died one hour after delivery. The diagnosis of bilateral renal agenesis was confirmed on autopsy.

Polish law prohibits termination of pregnancy after the second trimester. However, even when the decision to continue pregnancy is made, a precise diagnosis may help health-care professionals prepare the parents for the neonatal outcome and make a decision concerning postnatal management of the child.

## Discussion

Urinary tract malformations are quite common abnormalities, and are detectable with the use of obstetric sonography. Their incidence varies from 0.1% to 1% of all pregnancies [8]. The spectrum of these malformations is wide, from minor to severe and potentially lethal. Genitourinary tract abnormalities, including isolated anomalies and anomalies that suggest some particular congenital syndromes, comprise approximately 30% of antenatally detected anomalies [9]. Early diagnosis with ultrasound is possible in most cases, but some remain undetected until the third trimester [10,11]. Oligohydramnios, anhydramnios (common clinical complications associated with fetal urinary tract malformation) and maternal obesity may limit the diagnostic accuracy of ultrasound examination, making additional imaging methods potentially beneficial. A useful alternative tool to allow better visualization is amnioinfusion, but its invasive character exposes pregnant women to the possibility of complications such as premature rupture of membranes, amnionitis, fetal heart rate abnormalities or even embolisms [12]. MRI is currently accepted as a valuable technique for fetal anomalies assessment. The most common indications for performing fetal MRI for the assessment of the urinary tract system are oligohydramnios and anhydramnios. MRI is contraindicated in women with claustrophobia and relatively contraindicated in patients with metallic prostheses such as hip replacement implants and bone fracture fixation implants [13].

The suspicion of fetal urinary tract anomalies is a very commonly encountered indication for fetal MRI. Since many congenital syndromes are associated with the urinary tract, it should be investigated during every fetal MRI examination. During fetal development, the fetus keeps swallowing amniotic fluid and urine is continuously produced. A urine-filled bladder is an indirect sign of renal function. Since no urine is excreted, oligohydramnios arises. Due to lack of amniotic fluid, pulmonary maturation is severely impaired.

Renal development is a highly complex process and consists of three stages: the pronephros, mesonephros, and metanephros. Any disruptions at these early developmental stages can lead to renal agenesis. Bilateral renal agenesis has an incidence of 0.1 to 0.3 per 1000 births [14].

Fetal bilateral renal agenesis leads to perinatal death and therefore the diagnosis must be correct to avoid active labor procedures. Bilateral agenesis should be differentiated with ectopic location of the kidneys or hypoplastic kidneys. Bilateral renal agenesis, or hypogenesis, is part of a very severe congenital disorder called Potter’s syndrome [15]. Moreover, preexisting diabetes mellitus has been discussed as a possible cause of renal agenesis [16]. Davis *et al*. in their population-based case–control study suggest that the estimated risk of delivering a child with renal agenesis is over three times greater in mothers with diabetes compared to mothers without diabetes [17].

## Conclusions

USG remains the method of choice for routine prenatal screening. However, MRI plays an increasingly important role in the diagnosis of fetal abnormalities. MRI should be recommended whenever sonographic examination suggests bilateral renal agenesis but fails to provide a definite diagnosis. It is probable that fetal MRI soon will be routinely performed for certain fetal anomalies and therefore obstetricians should be familiar with this imaging method.

## Consent

Written informed consent was obtained from the patient for publication of this case report and any accompanying images. A copy of the written consent is available for review by the Editor-in-Chief of this journal.

## Abbreviations

MRI: Magnetic resonance; USG: Ultrasonography.

## Competing interests

The authors declare that they have no competing interests.

## Authors’ contributions

TG contributed to the study conception and design, and carried out the literature research and manuscript preparation. AKr carried out the literature research and assisted with the manuscript preparation. AS assisted with the manuscript preparation and manuscript review. AKw carried out the manuscript review and assisted with the literature research. TP assisted with the manuscript preparation and manuscript review. RP contributed to the study conception and design, carried out the literature research and manuscript preparation. All authors read and approved the final manuscript.
